# Off-Pump Coronary Revascularization Using Bilateral Internal Thoracic Arteries in A Patient with Paroxysmal Nocturnal Hemoglobinuria: A Case Report

**DOI:** 10.21470/1678-9741-2018-0071

**Published:** 2019

**Authors:** Juan Mariano Vrancic, Manuel Roque Cervetti, Julián Benavides, Daniel Navia

**Affiliations:** 1 Department of Cardiac Surgery, Instituto Cardiovascular de Buenos Aires, Buenos Aires, Argentina.

**Keywords:** Off-Pump Coronary Revascularization, Bilateral Internal Thoracic Arteries, Paroxysmal Nocturnal Hemoglobinuria

## Abstract

Paroxysmal nocturnal hemoglobinuria (PNH) is an ultra-orphan disease. We report the first case in the literature of Off-Pump Coronary Revascularization Using Bilateral Internal Thoracic Arteries in a patient with paroxysmal nocturnal hemoglobinuria.

A 36-year-old man came to the emergency department with acute non-ST elevation myocardial infarction (NSTEMI). He presented paroxysmal nocturnal hemoglobinuria diagnosed in 2016. Coronary angiography revealed tripple vessel disease.

The conduits used for coronary revascularization were both internal thoracic arteries (left ITA-right ITA [LITA-RITA]).

We consider that off-pump coronary artery bypass grafting (OPCABG) using Bilateral Internal Thoracic Arteries (BITA) can be safely performed with low in-hospital mortality and complications rates, even in patient with PNH.

**Table:** 

Abbreviations, acronyms & symbols	
BITA	= Bilateral internal thoracic arteries
DA	= Diagonal Artery
GPI	= Glycosyl-phosphatidylinositol
ICU	= Intensive Care Unit
ITA	= Internal thoracic arteries
LAD	= Left anterior descending
LCX	= Left circumflex coronary artery
LITA	= Left internal thoracic arteries
NSTEMI	= Non-ST elevation myocardial infarction
OPCABG	= Off-pump coronary artery bypass grafting
PIG-A	= phosphatidylinositol glycan, class A
PNH	= Paroxysmal nocturnal hemoglobinuria
RCA	= Right coronary artery
RITA	= Right internal thoracic arteries

## INTRODUCTION

Paroxysmal nocturnal heemoglobinuria (PNH), an ultra-orphan disease with a prevalence of 15.9 per million in Europe, is a life-threatening disorder, characterized by hemolysis, bone marrow failure and thrombosis^[1]^.

PNH is based on a clonal defect of hematopoietic stem cells characterized by deficiency in glycosyl-phosphatidylinositol (GPI)-anchored surface proteins due to mutations within the X-chromosomal PIG-A Gene (2).

PNH is an acquired hemolytic anemia associated with an increased risk to develop thrombocytopenia, atypical venous thrombosis and hypoplastic bone marrow^[2]^.

Nowadays, the use of new drugs such as Eculizumab (Soliris™) has improved the quality of life and the symptoms suffered by these patients.

To our knowledge,we report in the literature of off-pump coronary revascularization using bilateral internal thoracic arteries (BITA) in a patient with paroxysmal nocturnal hemoglobinuria.

## CASE REPORT

A 36-year-old man presented to the emergency department with an acute non-ST elevation myocardial infarction (NSTEMI) with elevated troponin.

Previous medical history included dilated cardiomyopathy with dyspnea, functional class II-III in study, and tripple vessel coronary disease. In addition, the patient presented paroxysmal nocturnal hemoglobinuria diagnosed in 2016, treated with eculizumab.

On admission, laboratory tests showed normal kidney function (blood urea nitrogen of 29mg/dl and creatinine of 0.93 mg/dl) and normal liver function. Leucocytes 4690/mm3. Differential blood count revealed 53.7% neutrophils, 33.7% lymphocytes, 11.1% monocytes, and 0.9% eosinophils. Hemoglobin 10.1 g/dl, platelets 113.000/mm^3^.

**Fig. 1:**
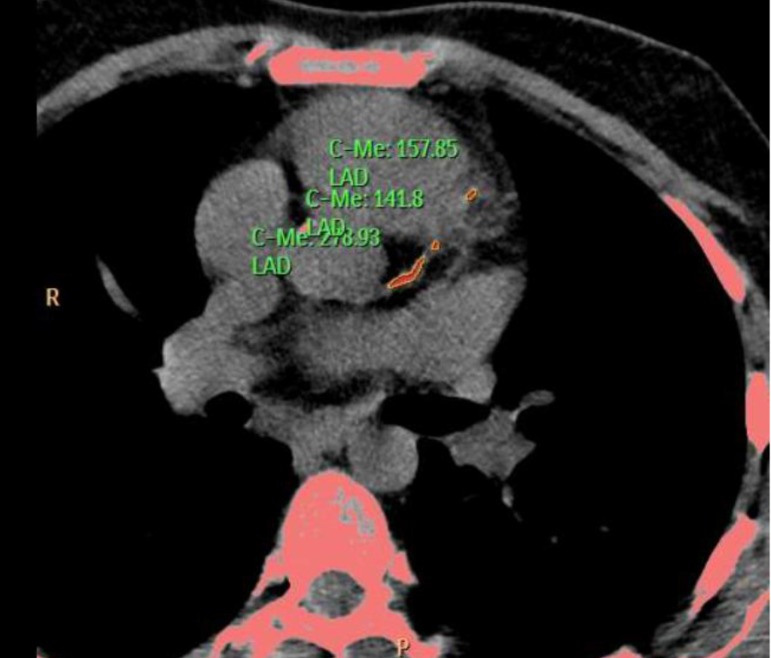
Coronary Angiotomography.

**Fig. 2:**
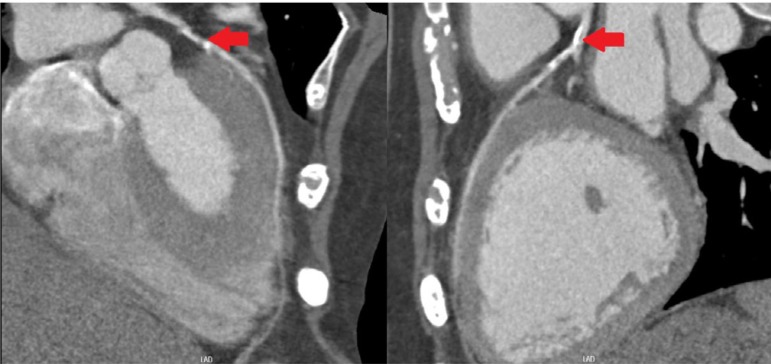
Anterior Descending Artery: Presents ostial and proximal lesion. Filled arrows indicate injuries.

**Fig. 3:**
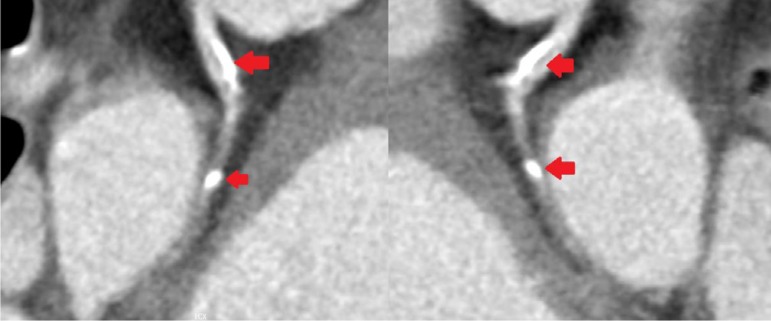
Circumflex: presents significant proximal stenosis by mixed lesion. Filled arrows indicate injuries.

**Fig. 4:**
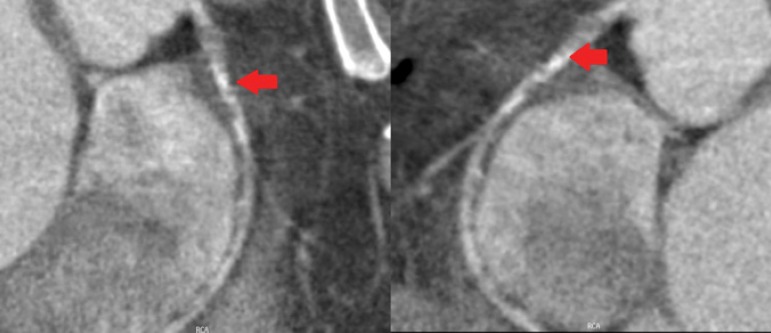
Coronary Right presents proximal and medium significant lesions. Filled arrows indicate injuries.

Coronary angiotomography reported the following: Left main presented a middle and distal lesion extended to the origin to the LAD; LAD with an ostial and proximal mixed lesion; Circunflex presented a significant proximal stenosis and right coronary is do inant and presented a very significant proximal and middle lesions. (Figures 1 to 4).

Coronary angiography revealed a moderate ostial and proximal stenoses in the left anterior descending (LAD) coronary artery. There were chronic stenoses in the proximal segment of the left circumflex coronary artery (LCX) and a 95% stenosis in the proximal right coronary artery.

Echocardiography showed reduced left ventricular function (Ejection fraction: 32%), severely dilated left ventricle and global hypokinesia.

The conduits used for coronary revascularization were both internal thoracic arteries (left ITA-right ITA [LITA-RITA]). The LITA was harvested and anastomosed to the left anterior descending artery. The technical configuration was in-situ anastomoses of the LITA to the left anterior descending artery; and the RITA, after being divided at its origin and bifurcation, was connected end to-side to the in-situ LITA as a sequential T graft to two circumflex arteries. Saphenous vein grafts were anastomosed to the posterior descending coronary artery. It was reported that skeletonized harvesting of ITA offers more conduit length and was associated with a lower incidence of sternal infection, so we use this technic routinely.

The procedure was off-pump, without complication.

During the postoperative period, subcutaneous heparin 5,000 units was administered 2 times daily, to avoid thromboembolism.

After 24 hours in the ICU and 4 days of uneventful total length of stay, the patient was discharged.

Eculizumab (900 mg) was administrated during the postoperative period to optimize hematological parameters.

The postoperative controls were at 7 days and at 25 days after discharge. The patient was asymptomatic.

## DISCUSSION

Thromboembolism is the most common cause of mortality in PNH, and the 4-year survival of patients presenting with thrombosis at diagnosis was only 40% before the era of eculizumab^[3]^. Arterial thromboembolism is far less frequent and only 20 cases of coronary thrombosis associated with PNH have been reported until now. Ziakas et al reported in a meta-analysis on 363 PNH cases with thrombosis, 12 episodes of myocardial infarction^[3,4]^.

Eculizumab is a humanized monoclonal antibody that blocks terminal complement pathway by binding to C5. This drug has dramatically changed the natural history of PNH. Eculizumab increases transfusion independency, reduces the risk of further thrombotic events and improves health-related quality of life. Eculizumab^[5]^.

Cardiac surgery in PNH patients is associated with several possible complications. PNH-induced granulocytopenia increases the risk of postoperative infection. The increase of hemolysis by extracorporeal circulation in cardiac surgery due to complement activation from either contact of blood with the foreign material surfaces during cardiopulmonary bypass circuit, or use of protamine to neutralize systemic heparin after cardiopulmonary bypass and tissue injury is well known^[2]^.

We consider that OPCABG using BITA can be safely performed with low in-hospital mortality and complications rates, even in patients with PNH. Surgical techniques and the new technology in coronary stabilizers allow surgeons to perform a complete myocardial coronary revascularization using the best available arterial conduit (BITA)^[6]^.

There have been 5 cases reported previously of patients with PNH undergoing cardiac surgery, but this is the first case where we combine Off-Pump Coronary Revascularization Using Bilateral Internal Thoracic Arteries in a patient with paroxysmal nocturnal hemoglobinuria.

**Table:** 

Author's roles & responsibilities	
JMV	Substantial contributions to the conception or design of the work; or the acquisition, analysis, or interpretation of data for the work; final approval of the version to be published
MRC	Substantial contributions to the conception or design of the work; or the acquisition, analysis, or interpretation of data for the work; final approval of the version to be published
JB	Substantial contributions to the conception or design of the work; or the acquisition, analysis, or interpretation of data for the work; final approval of the version to be published
DN	Substantial contributions to the conception or design of the work; or the acquisition, analysis, or interpretation of data for the work; final approval of the version to be published
